# St. George's and the Westminster Hospitals

**Published:** 1914-05-23

**Authors:** 


### The Hospital

The Workers' Newspaper of Administrative Medicine and Institutional Life, Administration, National Insurance and Health.

No. 1456, Vol. LVI. SATURDAY, MAY 23, 1914. PRICE ONE PENNY.

### ST. GEORGE'S AND THE WESTMINSTER HOSPITALS.

For several months, a longer period than the points to be determined would seem wholly to explain, negotiations between these hospitals for amalgamation have been talked about and debated. We have taken some trouble to ascertain wherein lies the true cause of the delays that have taken place, but can obtain no satisfactory answer. On the one hand there is a hospital where the business management is of the best, and on the other a hospital which has suffered and is suffering keenly from the want of business management in its affairs. Is it possible that an absence of business considerations and business knowledge may cause continuous delays in the case of one or both parties to these negotiations?

The position to-day is critical in the sense that it is essential that those parties to the negotiations who have business instincts should insist upon an end being put to the past delays, that a definite decision may be taken promptly. St. George's Hospital or some of its supporters were in favour at one time of a site at Wandsworth on the river, which it soon became apparent was neither high enough nor large enough, nor had it the requisite qualities of soil and hygienic considerations for the site of a new, modern, up-to-date hospital with 500 beds, or upwards, to which was attached an active and important medical school. This site was therefore declined, and although many efforts have been made to find a suitable alternative site on the north side of the river no suitable site has yet been discovered. There is the further question of the sale of the old sites to be provided for, and it is possible the absence of a purchaser may have delayed the decision.

On the other hand, Sir James Barry and his supporters at the Westminster Hospital have found a most attractive site facing south, overlooking Clapham Common and the country beyond, the view extending to the Sydenham Hills. It has the advantage also of being an island site with a total available area of twelve acres, which in our view is the minimum acreage requisite for a modern hospital for London containing 500 beds or upwards. Indeed, twelve acres are all too few; but the Clapham site has this further advantage, that the medical school buildings could be placed on an adjacent site so as to devote the whole twelve acres, if it be so decided, to hospital purposes solely. We understand that the Westminster authorities are ready and most anxious to proceed without further delay. What is the position and attitude of the authorities of St. George's Hospital on this point? It is time, in short, that a decision was taken, and that whatever its nature it should be made public without delay.

The sand in the hour-glass is running out fast. We may perhaps with advantage point out to the authorities of both hospitals that the members of the public resident in London who will be asked to provide the necessary funds, or most of them, for the erection of this great modern hospital of 500 beds, are not likely to sanction or support two schemes for two hospitals, each containing some 300 beds. That is why it is so essential to urge agreement and a common policy upon the authorities of both institutions. If they unite forces the proposition should not prove financially of much difficulty, but from the medical school point of view not to amalgamate means in practice that the hospital which is bold enough to take the best site and to build upon it, will secure the support of the majority of Londoners. After all, the people who can give large sums to hospitals for building purposes, even in London, are limited in numbers. Thanks to the King's Hospital Fund the most liberal supporters of hospitals do individually take at the present time great personal interest in all proposals which seriously affect the hospital accommodation provided under the voluntary system for the population of London. They know, as we have ventured to point out, that in the present case one of the two hospitals is controlled by men of business of the highest standing, who have given evidence of their capacity and competence. It must therefore be manifest that if the non-agreement of these two hospitals to a scheme of amalgamation should be proved to be caused by the attitude taken up by the other, the giving public will probably show their resentment by refusing to support it with funds. In other words, they may decline to sanction the removal of that hospital from its present site or the expenditure of large sums of money by its managers upon a scheme to erect a new and separate hospital and to retain a medical school in connection with it. If the scheme of amalgamation is to be consummated it must eventuate quickly and successfully.

